# Highly expressed carbohydrate sulfotransferase 11 correlates with unfavorable prognosis and immune evasion of hepatocellular carcinoma

**DOI:** 10.1002/cam4.5186

**Published:** 2022-09-05

**Authors:** Dan‐dan Xiong, Jian‐di Li, Rong‐quan He, Ming‐xuan Li, Yan‐qing Pan, Xiao‐lian He, Yi‐wu Dang, Gang Chen

**Affiliations:** ^1^ Department of Pathology First Affiliated Hospital of Guangxi Medical University Nanning China; ^2^ Department of Medical Oncology First Affiliated Hospital of Guangxi Medical University Nanning China

**Keywords:** CHST11, hepatocellular carcinoma, prognosis, Treg cells

## Abstract

Despite great advance has been made in multi‐modality treatments for HCC patients, the effectiveness is far from satisfactory with worse survival outcome, which may be partly explainable by the anti‐tumor deficiency of the immune system. It is necessary to clarify the molecular mechanism of HCC immunodeficiency. Here, we demonstrated that carbohydrate sulfotransferase 11 (CHST11) was upregulated in HCC and related to advanced TNM stage. HCC patients with TP53 mutation showed higher CHST11 expression. Survival analysis revealed that CHST11 was an independent prognostic biomarker in HCC. Cellular functional experiments indicated that knockdown of CHST11 in HCC inhibited cell proliferation and metastasis. Gene functional enrichment analyses indicated that CHST11 modulated pathways related to tumor growth, metastasis and immune regulation. Continuative immune‐related analyses revealed that CHST11 expression facilitated Tregs infiltration in HCC and promoted the expression of checkpoints PD‐L1/PD‐1, resulting in the immunosuppression of HCC. Targeting CHST11 may inhibit Tregs infiltration and enhance the antineoplastic effect of immune checkpoint inhibitors, which provides a novel insight into the combination immunotherapy with Treg‐modulating agents and PD‐L1/PD‐1 inhibitors.

## Graphical abstract

This is the first study to elucidate the relationship between CHST11 expression and prognosis as well as immune infiltration in HCC. These findings are potentially valuable in advancing our current understanding of CHST11 as a biomarker for HCC prognosis predicting and a molecular target for HCC immunotherapy.

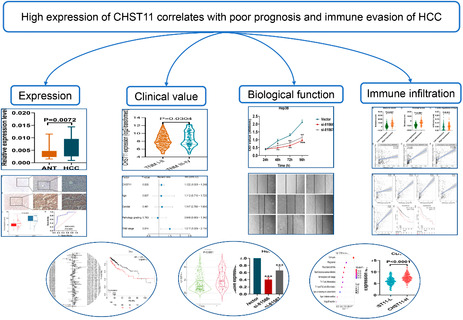

## 1 INTRODUCTION

Hepatocellular carcinoma (HCC) is one of the most frequently occurring malignances and ranks third in global tumor‐related mortality, with approximately 780,000 deaths and 840,000 new cases annually.^1^ Despite great advance has been made in the field of diagnosis and multi‐modality treatments, the effectiveness is far from satisfactory with poor prognosis for HCC patients, which is partly explained by the anti‐tumor deficiency of host immune system.^2^

With the increasing recognition of the role of immune system in tumor growth, researchers have focused on the immunotherapy in cancer therapy. Tumor immunotherapy aims to active host immune system to fight against cancer cells,^3^ and has shown obvious antineoplastic activity in the treatment of malignant tumors.^4^, ^5^, ^6^, ^7^ Due to the eximious efficacy, immunotherapy has been awarded the “Breakthrough of the Year” in 2013.^8^ Although immunotherapy has undoubtedly revolutionized treatment strategies for cancer patients, there is still a percentage of cases that fail to respond to immunotherapy, which may be caused by the complexity and diversity of the host tumor microenvironment (TME).^9^ Immune cells in human body constantly surveil the cells around them. Once an abnormality is found, they will immediately activate the immune system to eliminate the abnormal cells, such as foreign pathogens, damaged cells and tumor cells.^10^ However, cancer cells can develop escape strategies to bypass the host immunosurveillance. An immunosuppressive TME exerts a key role in providing an immune‐permissive environment for tumor growth, and regulatory T cells (Tregs) are important participants in the suppressive immune microenvironment, which lead to immunotherapy resistance.^11^ The core of this immunosuppressive environment for tumors is the activation of oncogenes and abnormal signaling pathways. Therefore, it is of great significance to clarify the key genes or signal pathways that regulate immunosuppressive TME in malignant tumors to improve the clinical outcome for tumor patients. Nevertheless, the molecular events that regulate immunosuppressive TME in HCC are not clear.

Carbohydrate sulfotransferase 11 (CHST11) encodes chondroitin 4‐sulfotransferase 1, a key enzyme involved in the synthesis of chondroitin sulfate glycosaminoglycans (CS).^12^ As a vital member of the extracellular matrix (ECM), CS interacts with various proteins and cell surface‐related ECM components in the TME to modulate tumor growth and metastasis.^13^ Given that limited evidence has illustrated the function and clinical significance of CHST11 in HCC, we conducted this study and integrated multiple evidences to investigate whether CHST11 is involved in HCC progression and immune regulation.

## 2 MATERIALS AND METHODS

### 2.1 Detection of CHST11 mRNA in HCC by in‐house RT‐qPCR

We collected 20 paired fresh‐frozen HCC samples and adjacent liver samples from patients with tumor resection before any preoperative therapy for real‐time quantitative polymerase chain reaction (RT‐qPCR) analysis. The cases were diagnosed by senior pathologists.

RNA extraction, RNA reverse transcription and RT‐qPCR experiment were executed with the manufactures' instruction. The primers for CHST11 (forward 5′‐CACAAGCCGTAAGCGGAGG‐3′; reverse 5′‐CATGGGGTCGCTGTACTTCC‐3′) and endogenous reference gene beta‐action (forward 5′‐TGGCACCCAGCACAATGAA‐3′; reverse 5′‐CTAAGTCATAGTCCGCCTAGAAGCA‐3′) were purchased from Sangon Biotech and Takara, respectively. The CT value of each sample was detected and CHST11 expression was determined with the 2^−ΔCT^ method. Paired *t*‐test enables comparison of difference between groups. A *p*‐value <0.05 indicates a statistical significance.

### 2.2 Validation of CHST11 mRNA in HCC by multi‐center datasets

HCC‐related gene microarrays and RNA‐sequencing expression profiling were obtained from Gene Expression Omnibus (GEO)^14^ with following search strategy: (“hepatocellular carcinoma” OR “HCC” OR “liver cancer”) AND (gene OR mRNA). Strict criteria for dataset inclusion were established: (I) patients should be diagnosed with HCC, (II) processed or raw expression data should be provided, (III) the expression values of CHST11 should be detected and (IV) the number of samples in each group should be at least three. Accordingly, the exclusion standards were as follows: (I) research objects were not human beings, (II) the expression data of CHST11 in HCC were unavailable and (III) the samples were duplicated. In addition, high‐throughput sequencing datasets were also collected from The Cancer Genome Atlas (TCGA)^15^ and International Cancer Genome Consortium (ICGC).^16^

The expression values of CHST11 in each included dataset were log2‐transformed. The overall standard mean deviation (SMD) was determined with STATA 12.0 (Stata Corporation). SMD >0 with 95% confidence interval (CI) not containing zero indicates that CHST11 is highly expressed in HCC, SMD ≤0 with 95%CI not containing zero indicates that CHST11 is low expressed in HCC. Publication bias was assessed with Begg's and Egger's tests.

### 2.3 Clinical significances CHST11 mRNA in HCC

Clinicopathologic and survival information of HCC patients were collected from TCGA. Independent T‐test was used to disclose the relation between CHST11 mRNA expression and clinicopathologic parameters (age, gender, pathology grading, TNM stage and TP53 mutation) of HCC. Univariate and multivariate COX analyses were applied to elucidate the prognosis prediction capability of CHST11 mRNA in HCC. The prognostic role of CHST11in HCC was corroborated by the Kaplan–Meier plotter.^17^

### 2.4 Expression of CHST11 protein in HCC

CHST11 protein expression was detected by immunohistochemistry (IHC) staining in 90 HCC and adjacent normal liver specimens, which were gained from the First Affiliated Hospital of Guangxi Medical University. The cases received no preoperative radio‐chemotherapy and were diagnosed pathologically after surgery. The rabbit polyclonal antibody of CHST11 (1:100 dilution) were purchased from Abcam. The experiment was executed with the manufacturer's instruction.

CHST11 protein is mainly located in cytoplasm. Two pathologists independently assess the staining intensity and percentage of CHST11 protein in 10 consecutive and representative fields (×400 magnification microscope) for each slice. The expression of CHST11 protein was assessed with the immunoreactive scores (IRS).^18^ Sample was determined as CHST11 high expression (IRS >6) or CHST11 low expression (IRS ≤6). Mann–Whitney U non‐parametric test was employed to compare the CHST11 protein expression between groups. A receiver operating characteristic (ROC) curve was generated to assess the capability of CHST11 protein to discriminate HCC samples from normal liver samples.

### 2.5 Clinical significances CHST11 protein in HCC

The clinical information of the 90 cases was presented in Table S1. Mann–Whitney U non‐parametric test was used to analyze the relationship between CHST11 expression and clinicopathological parameters of age, gender, tumor size, pathology grading and Barcelona Clinic Liver Cancer (BCLC) stage.

### 2.6 In vitro experiments

#### 2.6.1 Cell culture

HCC cells Huh7 and Hep3B were incubated in cell incubator at 37°C with DMEM medium containing 10% fetal bovine serum.

#### 2.6.2 Cell transfection

CHST11 small interfere RNA (siRNA) lentiviruses were purchased from Genechem. Two CHST11 siRNA lentiviruses (si‐81566 and si‐81567) and control vector were transfected into Huh7 and Hep3B cells according to the manufacture’ instruction. RT‐qPCR method was used to verify the knockdown efficiency.

#### 2.6.3 Cell functional experiments

CCK8 test was executed to measure the effect of CHST11 silencing on HCC cell viability according to the manufactures' protocol. Wound‐healing test was performed to evaluate the effect of CHST11 silencing on HCC cell migration according to the previous study.^19^

### 2.7 Collection of genes correlated to CHST11 in HCC

To obtain differently expressed genes (DEGs) in HCC, we collected data from TCGA and The Genotype‐Tissue Expression (GTEx).^20^ DEGs were calculated by *limma‐voom* in R package after removing of batch effect and identified with the threshold of |log2(fold change)| ≥ 1 and adjusted *p* < 0.05. Correlations of the DEGs and CHST11 in HCC were analyzed using Pearson correlation analysis. Genes with correlation coefficient >0.3 and *p* < 0.05 were regard as significantly correlated to CHST11.

### 2.8 Enrichment analyses

Gene Ontology analysis of biological process and Kyoto Encyclopedia of Genes and Genomes (KEGG) analysis were executed with R package *ClusterProfiler*. KEGG pathway was visualized with *pathview* package in R. Gene‐set enrichment analysis (GSEA) was conducted with GSEA 4.1.0 (BROAD Institute).

### 2.9 Tumor immune infiltration analyses

Immune and stromal scores of 371 HCC patients were computed with R package *estimate* based on data from TCGA, and the fractions of 22 infiltrating immune cells in individual HCC sample were assessed with *CIBERSORT* in R. Our study gauged the correlations between CHST11 expression and immune score, stromal score and immune cell subpopulations in patients with HCC. Considering that the CIBERSORT algorithm may suffer from statistical multicollinearity caused by the inclusion of highly correlated immune cell types, we employed the TIMER2 website^21^ to corroborate immunomodulatory role of CHST11 in HCC.

## 3 RESULTS

### 3.1 Expression of CHST11 mRNA in HCC

**FIGURE 1:**
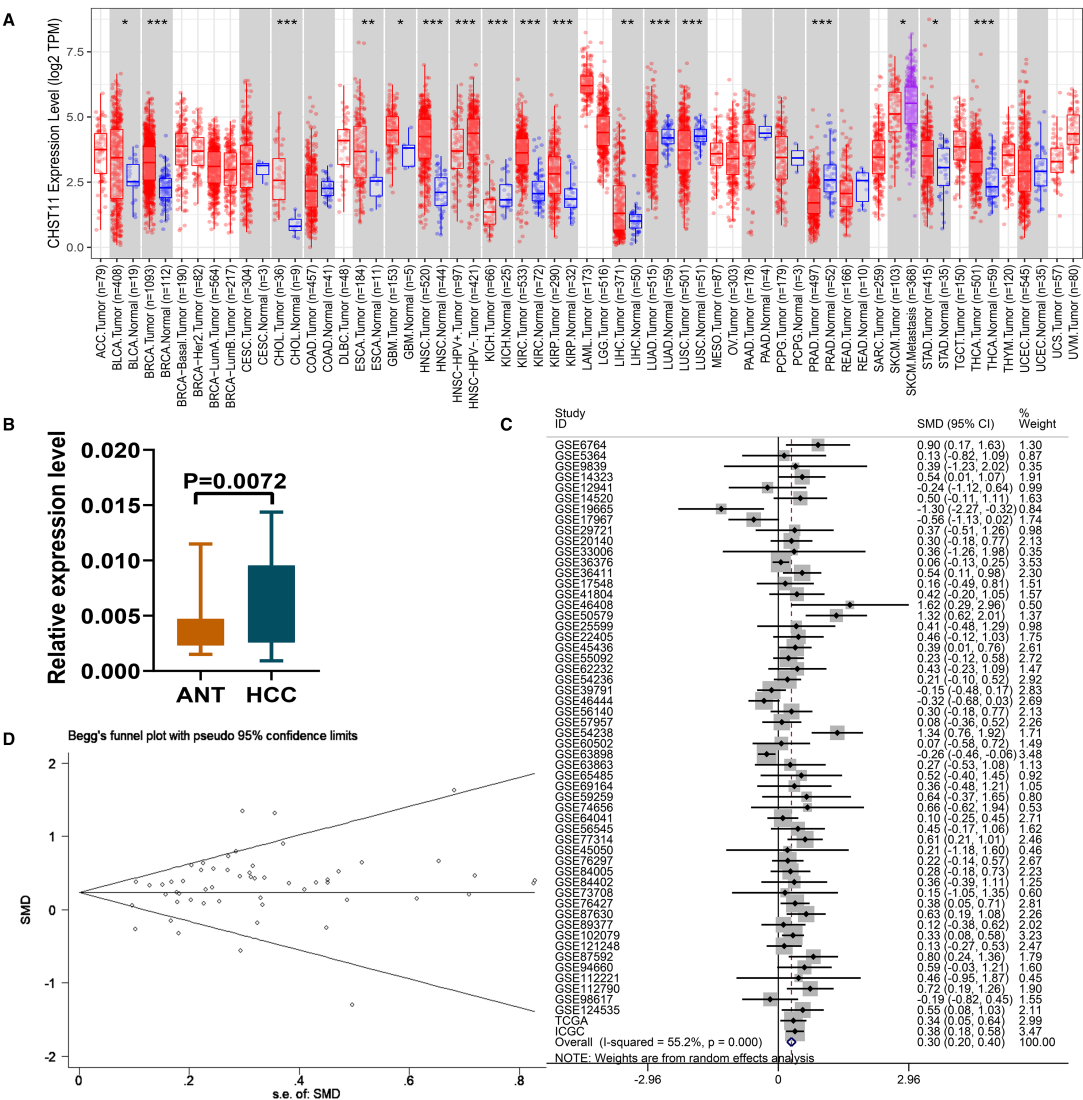
CHST11 mRNA expression level in human malignancies. (A) Expression of CHST11 mRNA in 33 cancers by the TCGA; (B) Expression of CHST11 mRNA in hepatocellular carcinoma (HCC) by RT‐qPCR; (C) Expression of CHST11 mRNA in HCC based on 56 multi‐center datasets; (D) Publication bias of 56 datasets included in this study. ANT, adjacent non‐tumor tissue.

CHST11 mRNA expression in human cancers was first analyzed using the TCGA RNA‐sequencing data. Increased expression of CHST11 mRNA was observed in bladder urothelial carcinoma, breast invasive carcinoma, cholangiocarcinoma, esophageal carcinoma, glioblastoma multiforme, head and neck squamous cell carcinoma, kidney renal clear cell carcinoma, kidney renal papillary cell carcinoma, liver hepatocellular carcinoma, stomach adenocarcinoma and Thyroid carcinoma (Figure 1A). Consistently, we corroborated the elevated expression of CHST11 mRNA in HCC by RT‐qPCR (*p* = 0.0072; Figure 1B). Simultaneously, we recruited 56 multi‐center datasets involving in 3110 HCC samples and 2016 non‐tumor samples from 12 countries around the world to further validate the expression of CHST11 mRNA in HCC. Figures [Link], [Link], [Link] presented the expression of CHST11 in the 56 datasets. Since the expression of CHST11 in individual datasets were not solid, we calculated the SMD based on the 56 datasets. A robust result showed that CHST11 was remarkably highly expressed in HCC tissues (SMD = 0.30, 95% CI = 0.20–0.40, *p* < 0.0001; Figure 1C), consistent with the RT‐qPCR. No publication bias existed in this study (Begg's *p* = 0.682, Egger's *p* = 0.645; Figure 1D).

### 3.2 Clinical significances of CHST11 mRNA in HCC

**FIGURE 2:**
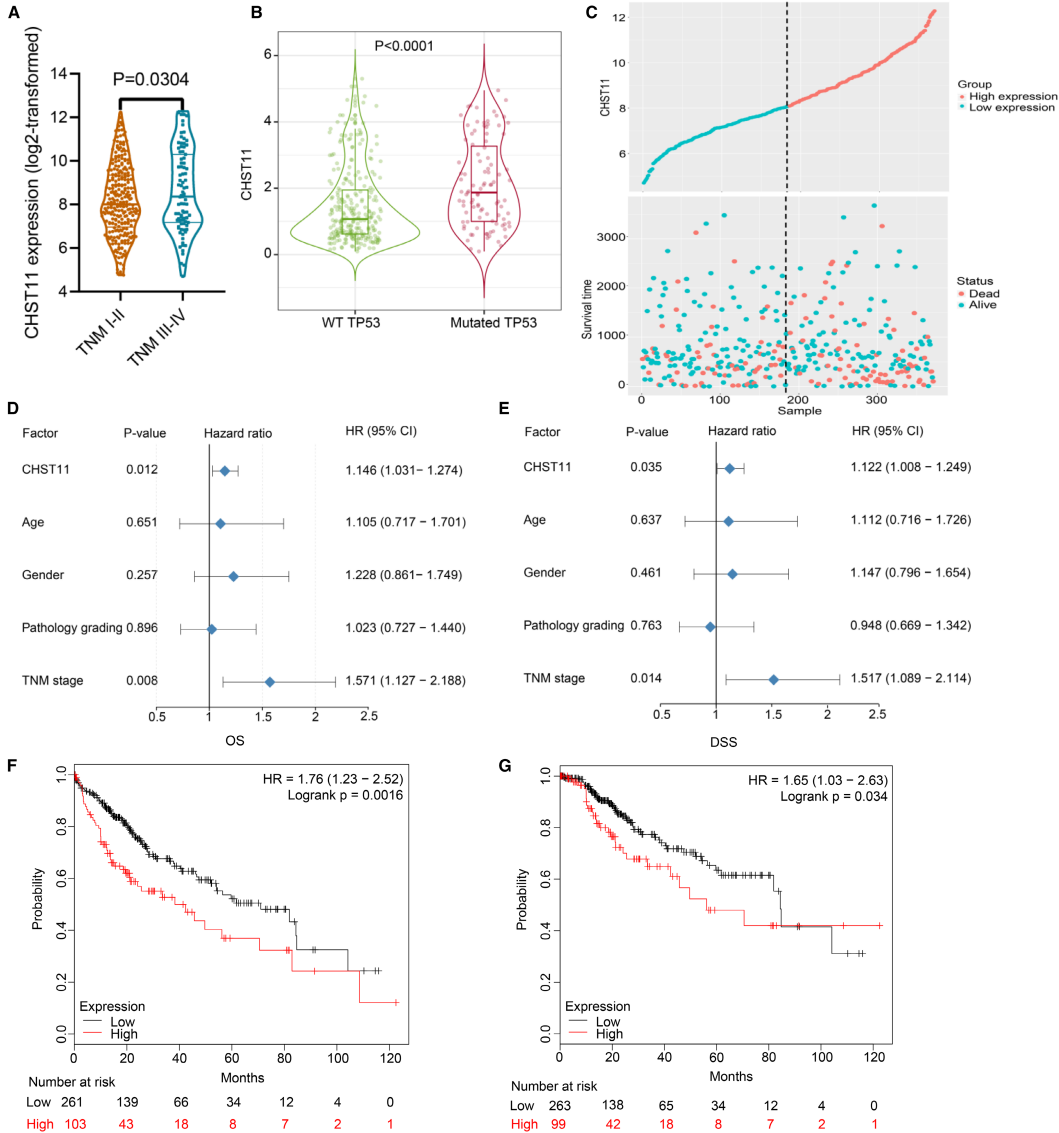
Clinical significances of CHST11 mRNA in hepatocellular carcinoma (HCC). Relationship of CHST11 mRNA expression with TNM stage (A) as well as TP53 mutation (B); (C) Survival status of HCC patients in high‐ and low‐ CHST11 expression groups; (D) Univariate COX analysis; (E) Multivariate COX analysis; Relationship of CHST11 mRNA expression with overall survival (F) and disease‐specific survival (G) in HCC by the Kaplan–Meier Plotter database.

CHST11 mRNA expression was related to TNM stage, but not to age, sex, or pathological grading in HCC. Patients with TNM stage III‐IV exhibited higher CHST11 mRNA expression than those with TNM stage I‐II (*p* = 0.0304; Figure 2A). Additionally, we assessed the association between CHST11 mRNA expression and TP53 mutation in 358 HCC samples, and found that CHST11 mRNA was highly expressed in mutated TP53 group than in wild‐type TP53 group (*p* < 0.0001; Figure 2B).

To assess the prognostic value of CHST11 in HCC, we collected 370 HCC patients with survival information from TCGA and divided them into high‐ and low‐CHST11 expression groups according to the median expression value of CHST11 (Figure 2C). Univariate COX analysis revealed that increased CHST11 mRNA expression and advanced TNM stage in HCC predicted worse survival outcome (CHST11: hazard ratio [HR] = 1.146, 95% CI = 1.031–1.274, *p* = 0.012; TNM stage: HR = 1.571, 95% CI = 1.127–2.188, *p* = 0.008; Figure 2D). Multivariate COX analysis further revealed that CHST11 mRNA and TNM stage were independent prognostic biomarkers in HCC (CHST11: HR = 1.122, 95% CI = 1.008–1.249, *p* = 0.035; TNM stage: HR = 1.517, 95% CI = 1.089–2.114, *p* = 0.014; Figure 2E). Furthermore, data from the Kaplan–Meier plotter validated that HCC patients with higher expression level of CHST11 showed shorter overall survival (HR = 1.76, 95% CI = 1.23–2.52, *p* = 0.0016; Figure 2F) as well as disease‐specific survival (HR = 1.65, 95% CI = 1.03–2.63, *p* = 0.034; Figure 2G).

### 3.3 Expression of CHST11 protein in HCC

**FIGURE 3:**
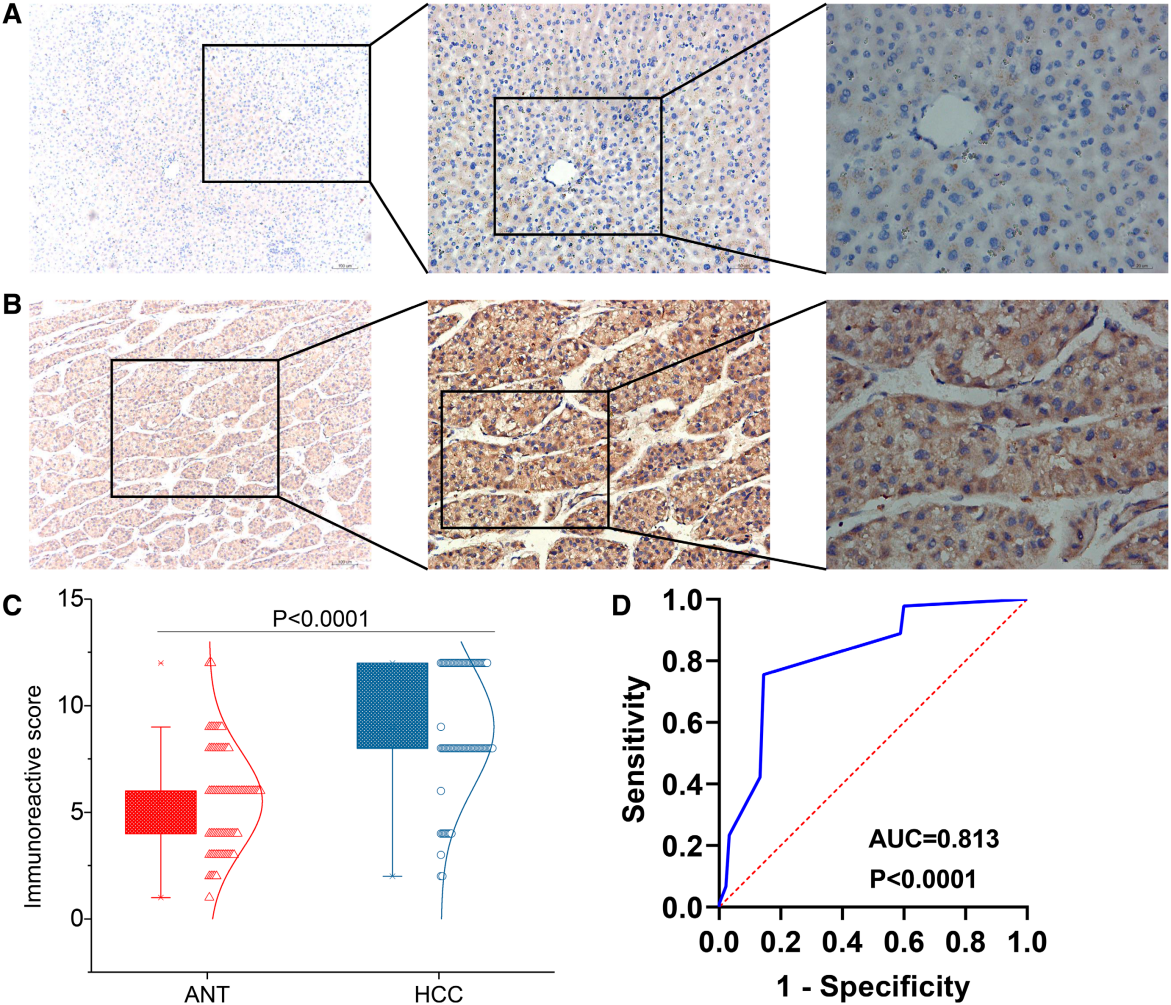
CHST11 protein expression pattern in hepatocellular carcinoma (HCC). Expression of CHST11 protein in adjacent non‐tumor (ANT) specimens (A) and HCC specimens (B) (magnification: ×40 (left), ×100 (middle) and ×200 (right)). CHST11 protein was mainly located at cell cytoplasm; (C) Box plot showing the expression of CHST11 protein in HCC and ANT specimens; (D) Receiver operating characteristic curve of CHST11 protein expression in HCC specimens and ANT specimens.

We scored CHST11 expression in the 90 HCC tissues and matched non‐tumor tissues with IRS system. In HCC group, 77 samples showed high CHST11 expression and 13 samples showed low CHST11expression, while in adjacent non‐tumor group, 21 samples showed high CHST11 expression and 69 samples showed low CHST11 expression. CHST11 protein expression was obviously increased in HCC specimens than in non‐tumor specimens (*Z* = 67.901, *p* < 0.0001; Figure 3A‐C and Figure S4). A ROC curve was generated with the area under the curve (AUC) value of 0.813 (95% CI = 0.749–0.877, *p* < 0.0001; Figure 3D), suggesting the capability of CHST11 to differentiate HCC specimens from non‐cancerous liver specimens.

### 3.4 Relationship between CHST11 expression and clinicopathological parameters in HCC

The relationship between CHST11 expression and clinicopathological parameters (age, gender, pathology grading and BCLC stage) in HCC has been investigated. However, the results showed no relationship between CHST11 expression and these clinicopathological characteristics (Table S1).

### 3.5 Effects of silenced CHST11 on HCC cell biological behaviors

**FIGURE 4:**
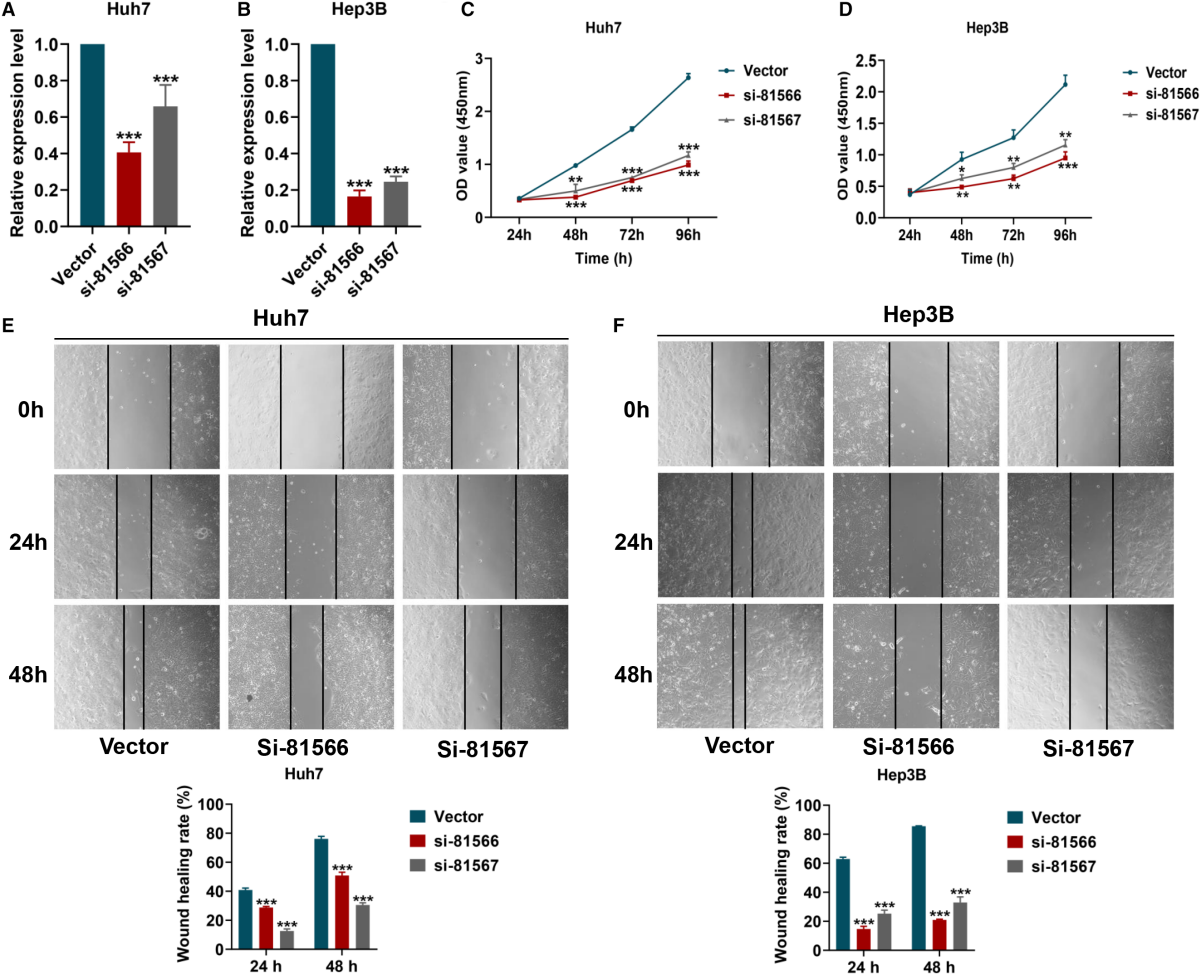
Effects of CHST11 knockdown on cell proliferation and metastasis in hepatocellular carcinoma (HCC). Knockdown of CHST11 in Huh7 cells (A) and Hep3B cells (B) with CHST11 silenced lentivirus; Effects of CHST11 knockdown on cell proliferation of Huh7 (C) and Hep3B (D) by CCK8 assay; Effects of CHST11 knockdown on cell metastasis of Huh7 (E) and Hep3B (F) by wound‐healing assay. *p < 0.05 **p < 0.01 ***p < 0.001.

CHST11 expression was significantly reduced in Huh7 and Hep3B cells transfected with CHST11 silenced lentivirus (si‐81566 and si‐81567) (Figure 4A‐B). As shown in Figure 4C‐F, the cell proliferation and wound healing rates in si‐81566 and si‐81567 groups were remarkably lower than that in vector group.

### 3.6 Functional enrichment analysis of genes correlated to CHST11 in HCC

**FIGURE 5:**
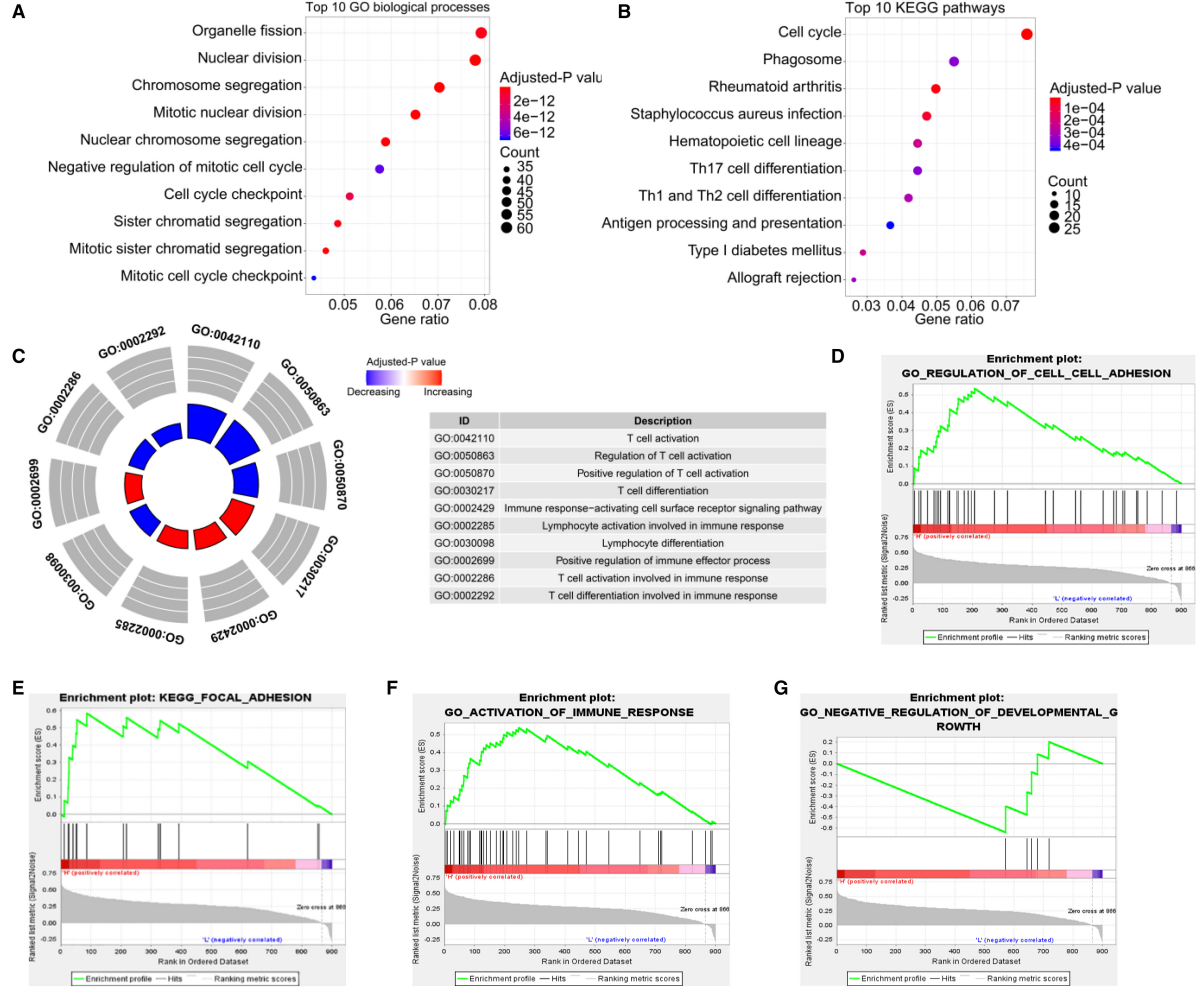
Functional enrichment analysis of genes correlated with CHST11 in hepatocellular carcinoma (HCC). (A) Top 10 GO biological progresses; (B) Top 10 KEGG pathways; (C) Immune‐related biological processes enriched by genes correlated with CHST11; (D) GSEA analysis showing GO term “regulation of cell‐cell adhesion” enriched by HCC samples with up‐regulated CHST11; (E) GSEA analysis showing KEGG term “focal adhesion” enriched by HCC samples with up‐regulated CHST11; (F) GSEA analysis showing GO term “activation of immune response” enriched by HCC samples with up‐regulated CHST11; (G) GSEA analysis showing GO term “negative regulation of development growth” enriched by HCC samples with down‐regulated CHST11.

Totally, 4251 DEGs in HCC were obtained, of which 900 genes were significantly correlated with CHST11 (Figure S5). Functional enrichment analysis demonstrated that these genes participated in cell growth‐ and metastasis‐related processes, such as “nuclear division”, “cell cycle checkpoint”, “cell cycle” and “extracellular matrix structural constituent” (Figure 5A‐B). Interesting, we also found that the 900 genes involved in immune‐related biological processes and pathways, such as “T cell activation”, “T cell differentiation” and “PD‐L1 expression and PD‐1 checkpoint pathway in cancer” (Figure 5C and Figure S6). Further GSEA analysis revealed that high expression of CHST11 positively regulated cell–cell adhesion (Figure 5D), focal adhesion (Figure 5E) and activation of immune response (Figure 5F), and low expression of CHST11 negatively regulated cell growth (Figure 5G).

### 3.7 Correlation of CHST11 expression and immune cell infiltration

**FIGURE 6:**
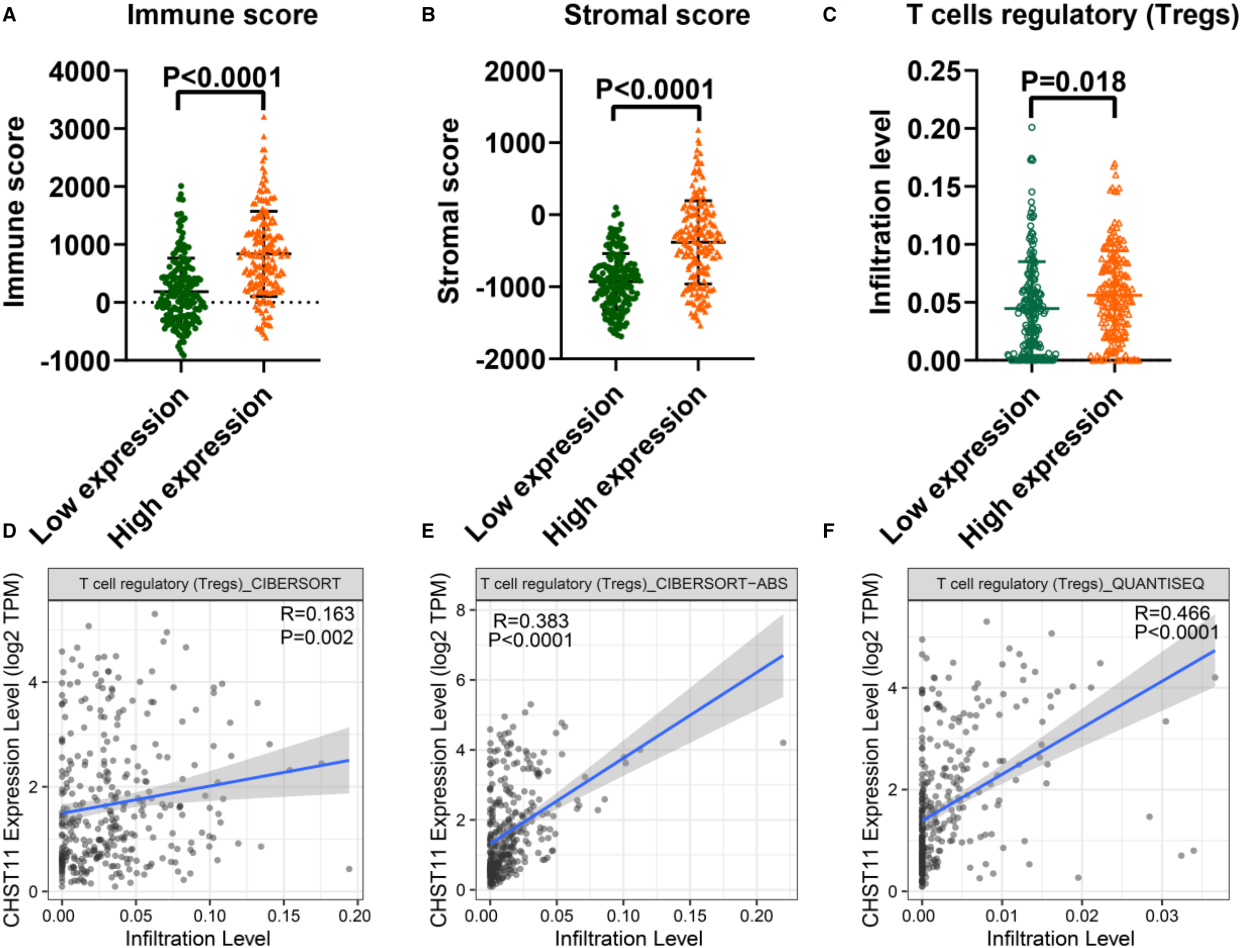
Effects of CHST11 expression on immune cell infiltration in hepatocellular carcinoma (HCC). (A) Higher immune score in high‐CHST11 expression group than in low‐CHST11 expression group in HCC; (B) Higher stromal score in high‐CHST11 expression group than in low‐CHST11 expression group in HCC; (C) Higher Tregs infiltration in high‐CHST11 expression group than in low‐CHST11 expression group in HCC with CIBERSORT algorithm; (D‐F) Positive relationship between CHST11 expression and Tregs infiltration in HCC by TIMER 2.0 website.

**FIGURE 7:**
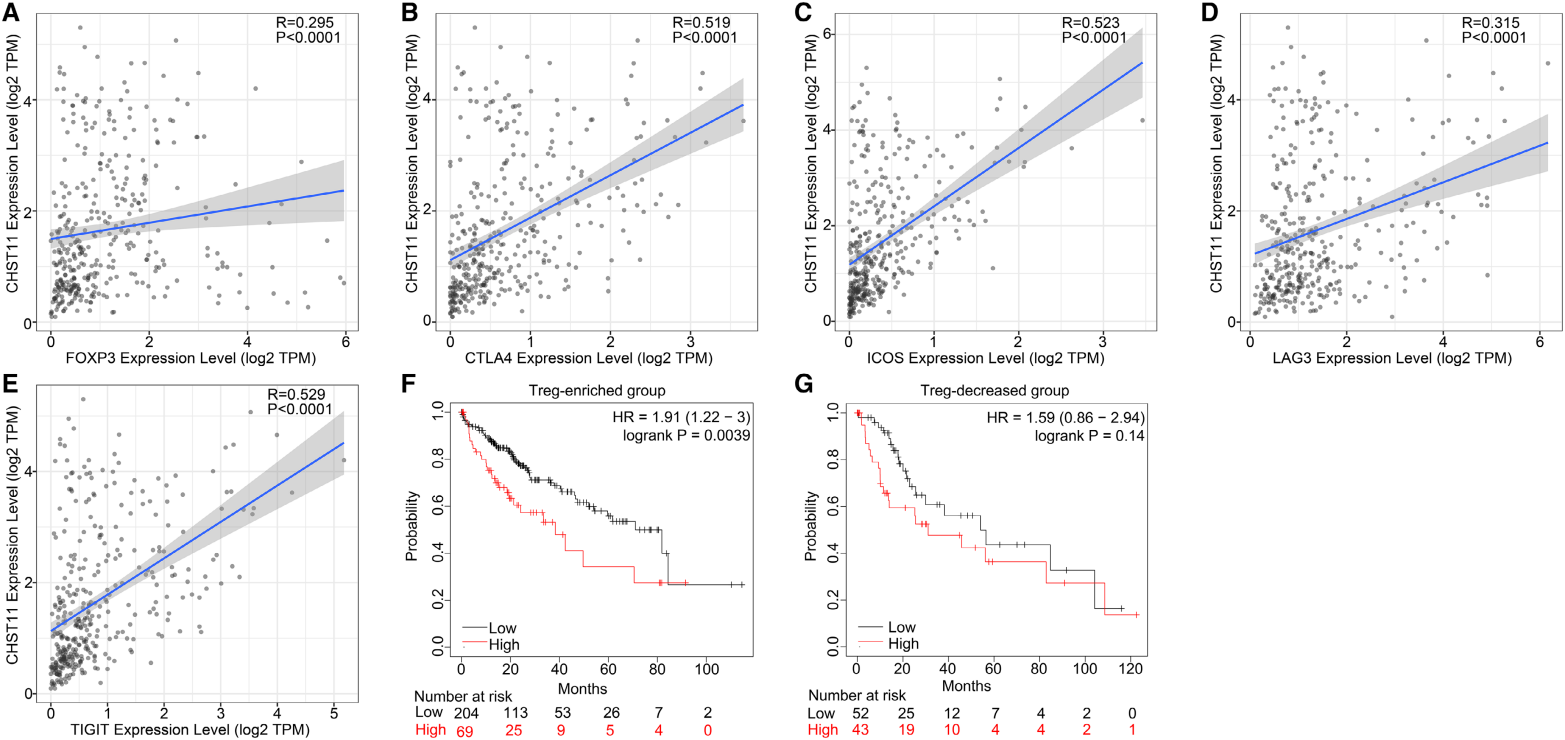
Relationship between CHST11 expression and TregS biomarkers in hepatocellular carcinoma (HCC). Correlation of CHST11 expression with FOXP3 (A), CTLA4 (B), ICOS (C), LAG3 (D) TIGIT (E). Prognostic value of CHST11 in Treg‐enriched (F) and Treg‐decreased (G) HCC patients.

TME is a complex milieu of tumor epithelial cells, immune cells, and stromal cells. To elucidate the regulatory role of CHST11 in TME in HCC, we gauged the relationship between CHST11 expression and immune cells as well as stromal cells. The results indicated that patients with increased CHST11 expression had higher immune score (*p* < 0.0001) and stromal score (*p* < 0.0001) compared to patients with decreased CHST11 expression (Figure 6A,B), suggesting more immune and stromal cells in patients with high CHST11 expression. Furthermore, we assessed the correlation between CHST11 expression and immune cell infiltration with CIBERSORT algorithm, and found that the infiltration of Tregs was higher in high‐CHST11 expression group than in low‐CHST11 expression group (*p* = 0.018; Figure 6C). The positive correlation between CHST11 expression and Tregs infiltration were corroborated by TIMER2 with algorithms of CIBERSORT (*R* = 0.163, *p* = 0.002; Figure 6D), CIBERSORT‐ABS (*R* = 0.383, *p* < 0.0001; Figure 6E), and Quantiseq (*R* = 0.466, *p* < 0.0001; Figure 6F). Simultaneously, we demonstrated that CHST11 expression was closely related to Treg cell intracellular and extracellular markers (FOXP3, CTLA4, ICOS, LAG3 and TIGIT) (Figure 7A‐E), which were involved in the activation or differentiation of Tregs. We divided HCC patients into Treg‐enriched and Treg‐decreased groups and found that in Treg‐enriched group, highly expressed CHST11 indicated adverse prognosis (HR = 1.91, 95% CI = 1.22–3, *p* = 0.0039; Figure 7F). However, in Treg‐decreased group, there was no significant correlation between CHST11 expression and prognosis (HR = 1.59, 95% CI = 0.86–2.54, *p* = 0.14; Figure 7G). These results revealed that the overexpressed CHST11 in HCC may be associated with the activation and differentiation of Tregs and thus lead to unfavorable prognosis of patients. Additionally, we also demonstrated that CHST11 expression was significantly positively correlated with immune checkpoints PD‐L1 and PD‐1 based on data from the TCGA and ICGC (Figure S7A‐D), which could be explained by the results of KEGG (Figure S6). According to the KEGG analysis, CHST11 may participate in PD‐L1 expression and PD‐1 checkpoint pathway in cancer by regulating genes TICAM2, LAT, TLR2, CD4, STAT3, NFKBIE and CD3D. All of these genes were positivity correlated with CHST11 (Figure S7E) and were overexpressed in high‐CHST11 expression group (Figure S7F‐L). The above evidences revealed the possible relationship between CHST11 and HCC immunosuppression, providing a novel therapeutic target for HCC immunotherapy.

## 4 DISCUSSION

Due to an insidious onset, most of HCC patients are diagnosed at an advanced stage, leading to a clinical resectable rate of <20%.^22^ For patients with inoperable HCC, the 5‐year survival rate is only about 8%,^23^ which may be partly due to tumor metastasis.^24^ The key prerequisite of tumor metastasis is the weakened cell adhesion ability, which is mainly regulated by adhesion‐related factors on the cell surface and in the ECM. Cell surface and ECM are rich in CS, which can be covalently combined with the core protein to form CS‐proteoglycan, thereby regulating cell adhesion and cell proliferation.^25^ CS can interact with some cell molecules (such as growth factors, adhesion molecules and cytokines) to affect disease progression.^26^ Increasing evidence has revealed that changing the structure and content of CS can promote tumor growth and enhance tumor invasion and metastasis.^27^ CS is synthesized in Golgi apparatus, and CHST11 is a key regulator for CS synthesis. Cooney et al.^28^ demonstrated that CS‐Proteoglycan regulated by CHST11 can be used as P‐selectin ligand to promote breast cancer metastasis. In addition, the carcinogenic effects of CHST11 in ovarian cancer,^29^ glioma,^13^ and leukemia^30^ have also been revealed. However, a study conducted by Kalathas et al.^31^ has presented a decreasing amount of CHST11 in colorectal cancer as the clinical stage of the cancer increases. The above evidences indicate that CHST11 plays a pro‐oncogenic or tumor‐suppressive role in tumor growth depending on cancer type. The role of CHST11 in HCC has not been fully understood.

This study demonstrated that CHST11 mRNA and protein both were highly expressed in HCC. The upregulated CHST11 was notably relevant to advanced TNM stage, TP53 mutation and unfavorable prognosis, suggesting that it could be served as a biomarker for HCC progression. Subsequent in vitro experiments revealed that silencing CHST11 inhibited HCC cell proliferation and metastasis. These evidences showed that CHST11 may exert a cancer‐promoting role in HCC. To uncover the potential mechanism of action of CHST11 in HCC progression, we collected genes those were significantly correlated with CHST11. Functional enrichment analyses indicated that these genes not only participated in tumor growth and metastasis‐related pathways, but also participated in immune‐related pathways, indicating the potential immune regulatory role of CHST11 in HCC.

Despite the recent advancements in cancer immunotherapy, its therapeutic effect in HCC is unsatisfactory. One main reason is that complicated TME developed multiple ways to promote tumor cells to escape host immune response.^32^ To clarify the immunological correlation between CHST11 expression and TME and the potential molecular mechanism of HCC immune evasion, we further conducted an immune infiltration analysis. We first found that immune cells and stromal cells were significantly elevated in tumor samples with high CHST11 expression. Further analyses revealed that increased CHST11 expression presented high immune infiltration of Tregs. Tregs are a naturally occurring T cell subset with immunosuppressive effects that can maintain immunologic balance in the body by inducing tolerance to self‐antigens.^33^ Yet in malignancies Tregs can secrete various inhibitory cytokines after being activated by TCR‐mediated signal stimulation to suppress host antitumor immunity.^34^ Increasing studies have shown that immune surveillance escape mediated by Tregs promotes the progression of malignant tumors.^35^ The number and proportion of Treg cells are abnormally increased in peripheral blood lymphocytes and tumor‐infiltrating lymphocytes in pancreatic cancer,^36^ breast carcinoma,^37^ gastric cancer,^38^ liver cancer^39^ and other malignant tumors.^40^ High Tregs ratio in the TME are associated with worse prognosis in majority of malignancies.^41^ Based on the above evidence, we speculated that CHST11 may regulate TME in HCC by promoting the infiltration of Tregs, thereby causing tumor cells to evade host immunity, further leading to tumor development and resulting in poor clinical outcomes. However, how CHST11 promotes Tregs infiltration remains unclear. Functional enrichment analyses indicted that CHST11 not only participated in biological processes related to T cell activation and differentiation, but also involved in the PD‐1/PD‐L1 signaling pathway. In addition, we also found that the expression of CHST11 was significantly positively correlated with PD‐1 and PD‐L1. Previous studies have demonstrated that the PD‐1/PD‐L1 pathway can induce the differentiation of Th1 cells into Tregs, promote the proliferation of Tregs, and maintain the immunosuppressive function of Tregs^.^ ^42^, ^43^ These findings suggested that CHST11 may promote Tregs activation and differentiation through the PD‐1/PD‐1 pathway, thereby regulating the immunosuppressive TME and promoting HCC progression.

At present, anti‐PD‐1/PD‐L1 antibodies have made major breakthroughs in tumor immunotherapy, but their efficacy on solid tumors is not good, and novel efficacy strategies for combination therapy need to be identified. The application of anti‐PD‐1/PD‐L1 antibodies can stimulate the proliferation and function of Tregs, thus mediating the occurrence of drug resistance in anti‐PD‐1/PD‐L1 therapy,^42^ but the specific mechanism of action is not yet fully understood. Here, we found that CHST11 was both involved in Tregs infiltration and the regulation of PD‐1/PD‐L1 pathway, and may be a potential combined target for anti‐PD‐1/PD‐L1 immunotherapy, providing a novel insight for improving the immunotherapy efficacy in solid tumors. However, there are limitations in this study. The immune‐related analysis of this study is based on RNA‐seq data and lack experimental validation. Next, we will validate the role of CHST11 in Tregs infiltration and anti‐PD‐1/PD‐LI immunotherapy via more clinical samples and further in vitro and in vivo studies.

In conclusion, the present study revealed that CHST11 facilitated tumor proliferation and metastasis of HCC. Highly expressed CHST11 may be interrelated to immunosuppression in HCC patients, resulting in poor prognosis. These findings are potentially valuable in advancing our current understanding of CHST11 as a marker for HCC prognosis predicting and a molecular target for HCC immunotherapy.

## AUTHOR CONTRIBUTION

XDD was mainly responsible for the experimental operations, data collection and manuscript writing. HRQ was mainly responsible for the data analysis and article revision. LJD, LMX, PYQ, HXL and DYW contributed to the data collection and figure plotting. CG contributed to the experiment supervision, study designation and manuscript revision.

## CONFLICT OF INTEREST

None.

## ETHICS STATEMENT

The Ethics Committee of the First Affiliated Hospital of Guangxi Medical University approved this study.

## PATIENT CONSENT STATEMENT

Not applicable.

## CLINICAL TRIAL REGISTRATION

Not applicable.

## Supporting information


Figure S1

Figure S2

Figure S3

Figure S4

Figure S5

Figure S6

Figure S7

Table S1

## Data Availability

The data that support the findings of this study are available from the corresponding author upon reasonable request.

## References

[1] Bray F , Ferlay J , Soerjomataram I , Siegel RL , Torre LA , Jemal A . Global cancer statistics 2018: GLOBOCAN estimates of incidence and mortality worldwide for 36 cancers in 185 countries. CA: Cancer J Clin. 2018;68(6):394‐424. 30207593 10.3322/caac.21492

[2] Bodzin AS , Busuttil RW . Hepatocellular carcinoma: Advances in diagnosis, management, and long term outcome. World J Hepatol. 2015;7(9):1157‐1167. 26019732 10.4254/wjh.v7.i9.1157 PMC4438491

[3] Shen Y , Liu L , Wang M , et al. TET2 inhibits PD‐L1 gene expression in breast cancer cells through histone deacetylation. Cancer. 2021;13(9):2207. 10.3390/cancers13092207 PMC8125390 34064441

[4] Bagchi S , Yuan R , Engleman EG . Immune checkpoint inhibitors for the treatment of cancer: Clinical impact and mechanisms of response and resistance. Annu Rev Pathol. 2021;16:223‐249. 33197221 10.1146/annurev-pathol-042020-042741

[5] Nasser NJ , Gorenberg M , Agbarya A . First line immunotherapy for non‐small cell lung cancer. Pharmaceuticals. 2020;13(11):373. 33171686 10.3390/ph13110373 PMC7695295

[6] Barata P , Hatton W , Desai A , et al. Outcomes with first‐line PD‐1/PD‐L1 inhibitor monotherapy for metastatic renal cell carcinoma (mRCC): A multi‐institutional cohort. Front Oncol. 2020;10:581189. 33194712 10.3389/fonc.2020.581189 PMC7642690

[7] Tsaur I , Brandt MP , Juengel E , Manceau C , Ploussard G . Immunotherapy in prostate cancer: New horizon of hurdles and hopes. World J Urol. 2021;39(5):1387‐1403. 33106940 10.1007/s00345-020-03497-1 PMC8514362

[8] Akkin S , Varan G , Bilensoy E . A review on cancer immunotherapy and applications of nanotechnology to chemoimmunotherapy of different cancers. Molecules. 2021;26(11):3382. 34205019 10.3390/molecules26113382 PMC8199882

[9] Chen X , Pan X , Zhang W , et al. Epigenetic strategies synergize with PD‐L1/PD‐1 targeted cancer immunotherapies to enhance antitumor responses. Acta Pharmaceutica Sinica B. 2020;10(5):723‐733. 32528824 10.1016/j.apsb.2019.09.006 PMC7276686

[10] Jiang X , Wang J , Deng X , et al. The role of microenvironment in tumor angiogenesis. J Exp Clin Cancer Res: CR. 2020;39(1):204. 32993787 10.1186/s13046-020-01709-5 PMC7526376

[11] Wang H , Franco F , Ho PC . Metabolic regulation of Tregs in cancer: Opportunities for immunotherapy. Trends Cancer. 2017;3(8):583‐592. 28780935 10.1016/j.trecan.2017.06.005

[12] Kluppel M . The roles of chondroitin‐4‐sulfotransferase‐1 in development and disease. Prog Mol Biol Transl Sci. 2010;93:113‐132. 20807643 10.1016/S1877-1173(10)93006-8

[13] Pan H , Xue W , Zhao W , Schachner M . Expression and function of chondroitin 4‐sulfate and chondroitin 6‐sulfate in human glioma. FASEB J. 2020;34(2):2853‐2868. 31908019 10.1096/fj.201901621RRR

[14] Barrett T , Wilhite SE , Ledoux P , et al. NCBI GEO: Archive for functional genomics data sets‐‐update. Nucleic Acids Res. 2013;41(Database issue):D991‐D995. 23193258 10.1093/nar/gks1193 PMC3531084

[15] Tomczak K , Czerwinska P , Wiznerowicz M . The cancer genome atlas (TCGA): An immeasurable source of knowledge. Contemporary Oncology. 2015;19(1A):A68‐A77. 25691825 10.5114/wo.2014.47136 PMC4322527

[16] Zhang J , Bajari R , Andric D , et al. The international cancer genome consortium data portal. Nat Biotechnol. 2019;37(4):367‐369. 30877282 10.1038/s41587-019-0055-9

[17] Gyorffy B . Survival analysis across the entire transcriptome identifies biomarkers with the highest prognostic power in breast cancer. Comput Struct Biotechnol J. 2021;19:4101‐4109. 34527184 10.1016/j.csbj.2021.07.014 PMC8339292

[18] Li Z , Pan W , Shen Y , et al. IGF1/IGF1R and microRNA let‐7e down‐regulate each other and modulate proliferation and migration of colorectal cancer cells. Cell Cycle. 2018;17(10):1212‐1219. 29886785 10.1080/15384101.2018.1469873 PMC6110595

[19] Xiong DD , Feng ZB , Lai ZF , et al. High throughput circRNA sequencing analysis reveals novel insights into the mechanism of nitidine chloride against hepatocellular carcinoma. Cell Death Dis. 2019;10(9):658. 31506425 10.1038/s41419-019-1890-9 PMC6737102

[20] Consortium GT . The genotype‐tissue expression (GTEx) project. Nat Genet. 2013;45(6):580‐585. 23715323 10.1038/ng.2653 PMC4010069

[21] Li T , Fu J , Zeng Z , et al. TIMER2.0 for analysis of tumor‐infiltrating immune cells. Nucleic Acids Res. 2020;48(W1):W509‐W514. 32442275 10.1093/nar/gkaa407 PMC7319575

[22] Qu Z , Jiang Y , Li H , Yu DC , Ding YT . Detecting abnormal methylation of tumor suppressor genes GSTP1, P16, RIZ1, and RASSF1A in hepatocellular carcinoma and its clinical significance. Oncol Lett. 2015;10(4):2553‐2558. 26622888 10.3892/ol.2015.3536 PMC4580012

[23] Ocker M , Mayr C , Kiesslich T , Stintzing S , Neureiter D . Immunmodulatory treatment strategies of hepatocellular carcinoma: From checkpoint inhibitors now to an integrated approach in the future. Cancer. 2021;13(7):1558. 10.3390/cancers13071558 PMC8036419 33805268

[24] Cheng X , Shi JB , Liu H , et al. Discovery of (4‐bromophenyl)(3‐hydroxy‐4‐methoxyphenyl)methanone through upregulating hTERT induces cell apoptosis and ERS. Cell Death Dis. 2017;8(8):e3016. 28837145 10.1038/cddis.2017.384 PMC5596570

[25] Farkas SA , Sorbe BG , Nilsson TK . Epigenetic changes as prognostic predictors in endometrial carcinomas. Epigenetics. 2017;12(1):19‐26. 27874289 10.1080/15592294.2016.1252891 PMC5270631

[26] Hayes A , Sugahara K , Farrugia B , Whitelock JM , Caterson B , Melrose J . Biodiversity of CS‐proteoglycan sulphation motifs: Chemical messenger recognition modules with roles in information transfer, control of cellular behaviour and tissue morphogenesis. Biochem J. 2018;475(3):587‐620. 29439148 10.1042/BCJ20170820

[27] Pudelko A , Wisowski G , Olczyk K , Kozma EM . The dual role of the glycosaminoglycan chondroitin‐6‐sulfate in the development, progression and metastasis of cancer. FEBS J. 2019;286(10):1815‐1837. 30637950 10.1111/febs.14748 PMC6850286

[28] Cooney CA , Jousheghany F , Yao‐Borengasser A , et al. Chondroitin sulfates play a major role in breast cancer metastasis: A role for CSPG4 and CHST11 gene expression in forming surface P‐selectin ligands in aggressive breast cancer cells. Breast Cancer Res. 2011;13(3):R58. 21658254 10.1186/bcr2895 PMC3218947

[29] Oliveira‐Ferrer L , Hessling A , Trillsch F , Mahner S , Milde‐Langosch K . Prognostic impact of chondroitin‐4‐sulfotransferase CHST11 in ovarian cancer. Tumour Biol. 2015;36(11):9023‐9030. 26084610 10.1007/s13277-015-3652-3

[30] Schmidt HH , Dyomin VG , Palanisamy N , et al. Deregulation of the carbohydrate (chondroitin 4) sulfotransferase 11 (CHST11) gene in a B‐cell chronic lymphocytic leukemia with a t(12;14)(q23;q32). Oncogene. 2004;23(41):6991‐6996. 15273723 10.1038/sj.onc.1207934

[31] Kalathas D , Theocharis DA , Bounias D , Kyriakopoulou D , Papageorgakopoulou N , Stavropoulos MS , Vynios DH . Alterations of glycosaminoglycan disaccharide content and composition in colorectal cancer: Structural and expressional studies. Oncol Rep 2009; 22(2):369–375. 19578779

[32] Kagedal A , Hjalmarsson E , Farrajota Neves da Silva P , et al. Activation of T helper cells in sentinel node predicts poor prognosis in oral squamous cell carcinoma. Sci Rep. 2020;10(1):22352. 33339891 10.1038/s41598-020-79273-3 PMC7749121

[33] Dong H , Qu S , Chen X , Zhu H , Tai X , Pan J . Changes in the cytokine expression of peripheral Treg and Th17 cells in children with rotavirus enteritis. Exp Ther Med. 2015;10(2):679‐682. 26622374 10.3892/etm.2015.2511 PMC4509078

[34] Takeuchi Y , Nishikawa H . Roles of regulatory T cells in cancer immunity. Int Immunol. 2016;28(8):401‐409. 27160722 10.1093/intimm/dxw025 PMC4986235

[35] Tanaka A , Sakaguchi S . Targeting Treg cells in cancer immunotherapy. Eur J Immunol. 2019;49(8):1140‐1146. 31257581 10.1002/eji.201847659

[36] Wang X , Wang L , Mo Q , Dong Y , Wang G , Ji A . Changes of Th17/Treg cell and related cytokines in pancreatic cancer patients. Int J Clin Exp Pathol. 2015;8(5):5702‐5708. 26191284 PMC4503155

[37] Clark NM , Martinez LM , Murdock S , et al. Regulatory T cells support breast cancer progression by opposing IFN‐gamma‐dependent functional reprogramming of myeloid cells. Cell Rep. 2020;33(10):108482. 33296659 10.1016/j.celrep.2020.108482 PMC7811278

[38] Ma K , Li X , Lv J , et al. Correlations between CD4(+) FoxP3(+) Treg and expression of FoxM1 and Ki‐67 in gastric cancer patients. Asia Pac J Clin Oncol. 2021;17(2):e63‐e69. 31957250 10.1111/ajco.13302

[39] Ou X , Guan J , Chen JS , et al. LAP(+)CD4(+) T cells are elevated among the peripheral blood mononuclear cells and tumor tissue of patients with hepatocellular carcinoma. Exp Ther Med. 2018;16(2):788‐796. 30116333 10.3892/etm.2018.6229 PMC6090257

[40] Peng L , Kjaergaard J , Plautz GE , et al. Tumor‐induced L‐selectinhigh suppressor T cells mediate potent effector T cell blockade and cause failure of otherwise curative adoptive immunotherapy. J Immunol. 2002;169(9):4811‐4821. 12391191 10.4049/jimmunol.169.9.4811

[41] Kalathil SG , Hutson A , Barbi J , Iyer R , Thanavala Y . Augmentation of IFN‐gamma+ CD8+ T cell responses correlates with survival of HCC patients on sorafenib therapy. JCI Insight. 2019;4(15):e130116. 31391334 10.1172/jci.insight.130116 PMC6693832

[42] Stathopoulou C , Gangaplara A , Mallett G , Flomerfelt FA , Liniany LP , Knight D , Samsel LA , Berlinguer‐Palmini R , Yim JJ , Felizardo TC , Eckhaus MA , Edgington‐Mitchell L , Martinez‐Fabregas J , Zhu J , Fowler DH , van Kasteren SI , Laurence A , Bogyo M , Watts C , Shevach EM , Amarnath S . PD‐1 inhibitory receptor downregulates asparaginyl endopeptidase and maintains Foxp3 transcription factor stability in induced regulatory T cells. Immunity 2018;49(2):247‐263.e7. 30054205 10.1016/j.immuni.2018.05.006 PMC6105434

[43] Park HJ , Park JS , Jeong YH , et al. PD‐1 upregulated on regulatory T cells during chronic virus infection enhances the suppression of CD8+ T cell immune response via the interaction with PD‐L1 expressed on CD8+ T cells. J Immunol. 2015;194(12):5801‐5811. 25934860 10.4049/jimmunol.1401936

