# Correction to “Highly Expressed Carbohydrate Sulfotransferase 11 Correlates With Unfavorable Prognosis and Immune Evasion of Hepatocellular Carcinoma”

**DOI:** 10.1002/cam4.70918

**Published:** 2025-05-01

Xiong DD, Li JD, He RQ, Li MX, Pan YQ, He XL, Dang YW, Chen G. Highly expressed carbohydrate sulfotransferase 11 correlates with unfavorable prognosis and immune evasion of hepatocellular carcinoma. Cancer Med. 2023;12:4938–4950. https://doi.org/10.1002/cam4.5186

In the “ACKNOWLEDGEMENTS” section, the grant number “NSFC82160862” was incorrect. The correct grant number is “NSFC82160762”.

We apologize for this error.

