# Supplementary figures and images for: Highly expressed carbohydrate sulfotransferase 11 correlates with unfavorable prognosis and immune evasion of hepatocellular carcinoma
Source: Cancer Med. 2022 Sep 5;12(4):4938–50. doi: 10.1002/cam4.5186 (PMC9972111; doi:10.1002/cam4.5186)

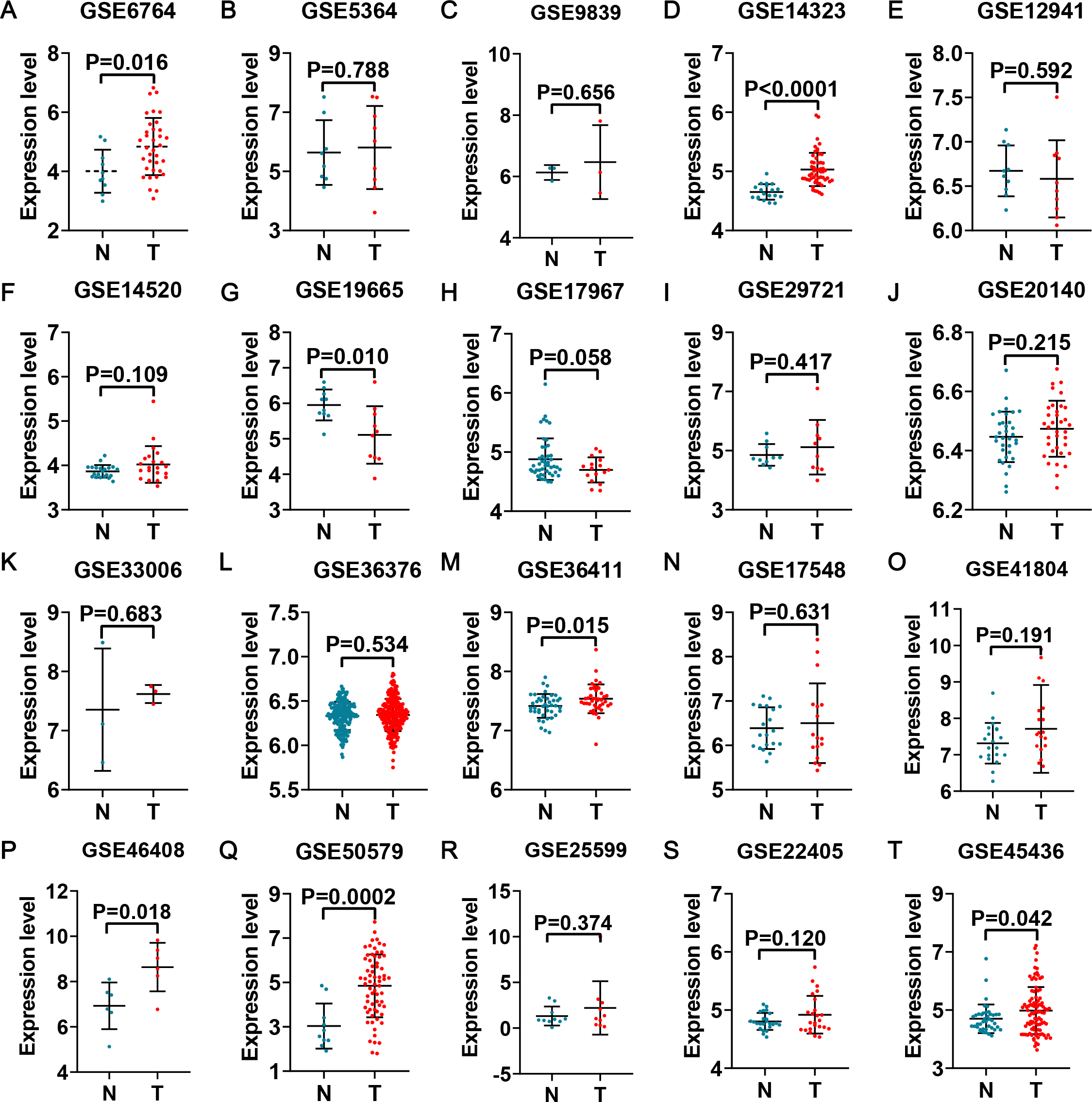

Supplement: Supplementary file 1 — Figure S1 [file CAM4-12-4938-s003.tif]

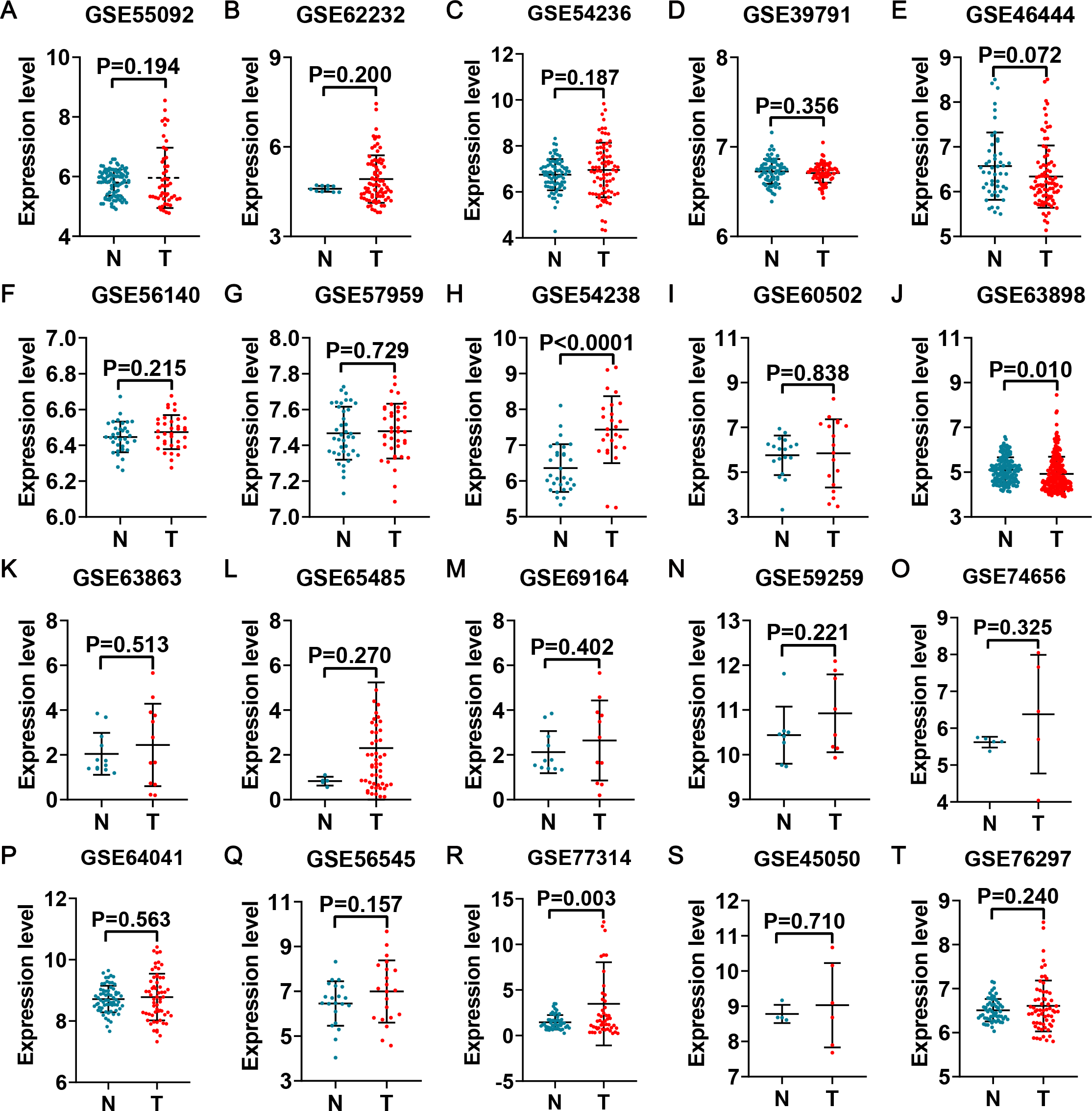

Supplement: Supplementary file 2 — Figure S2 [file CAM4-12-4938-s002.tif]

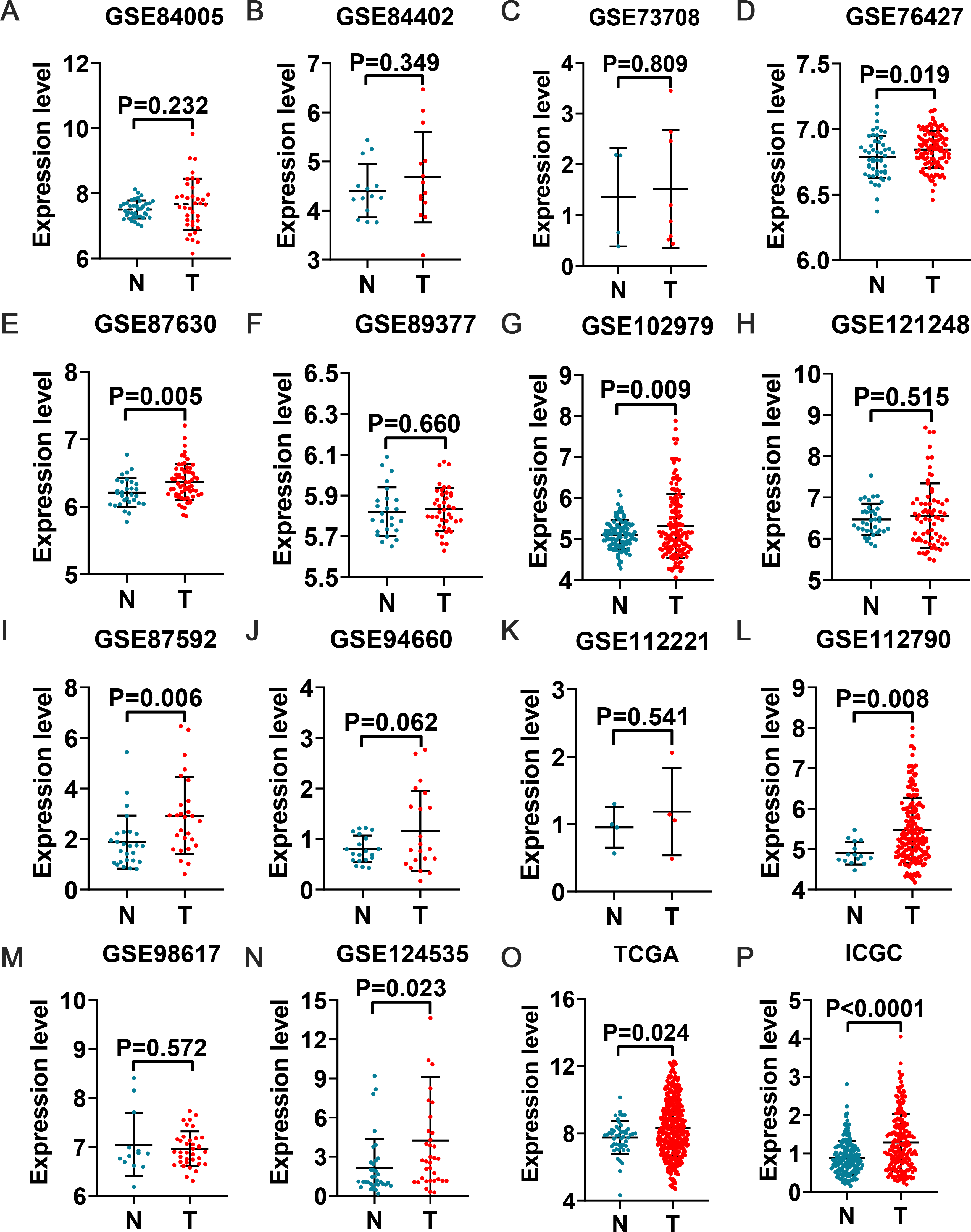

Supplement: Supplementary file 3 — Figure S3 [file CAM4-12-4938-s006.tif]

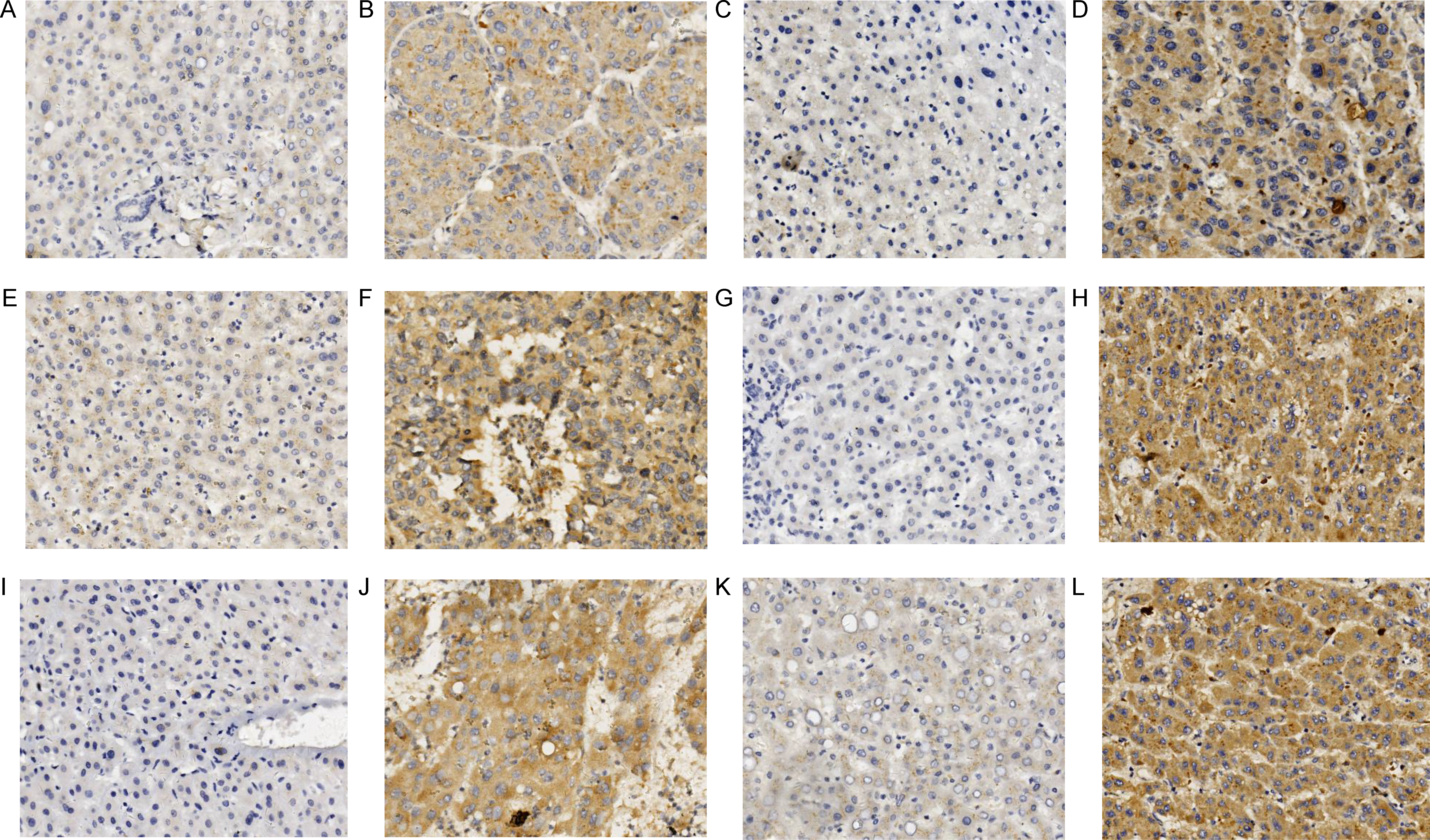

Supplement: Supplementary file 4 — Figure S4 [file CAM4-12-4938-s004.tif]

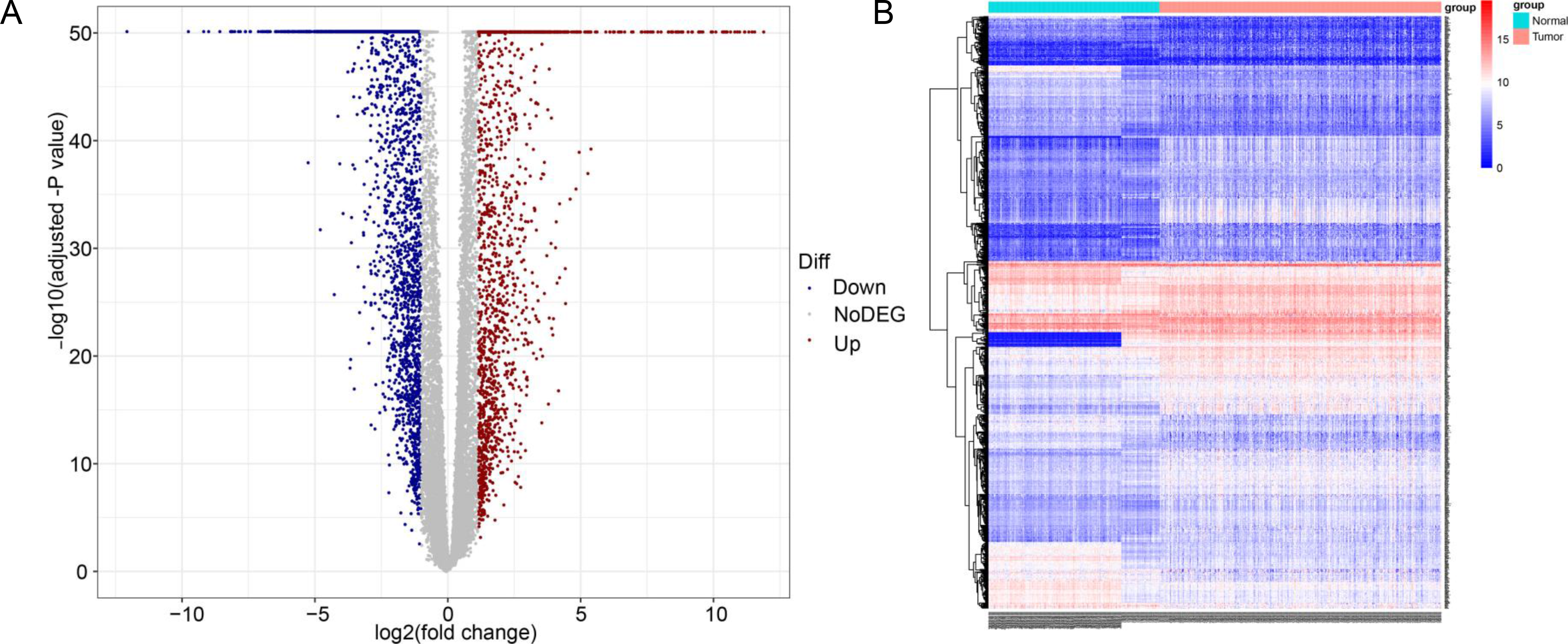

Supplement: Supplementary file 5 — Figure S5 [file CAM4-12-4938-s008.tif]

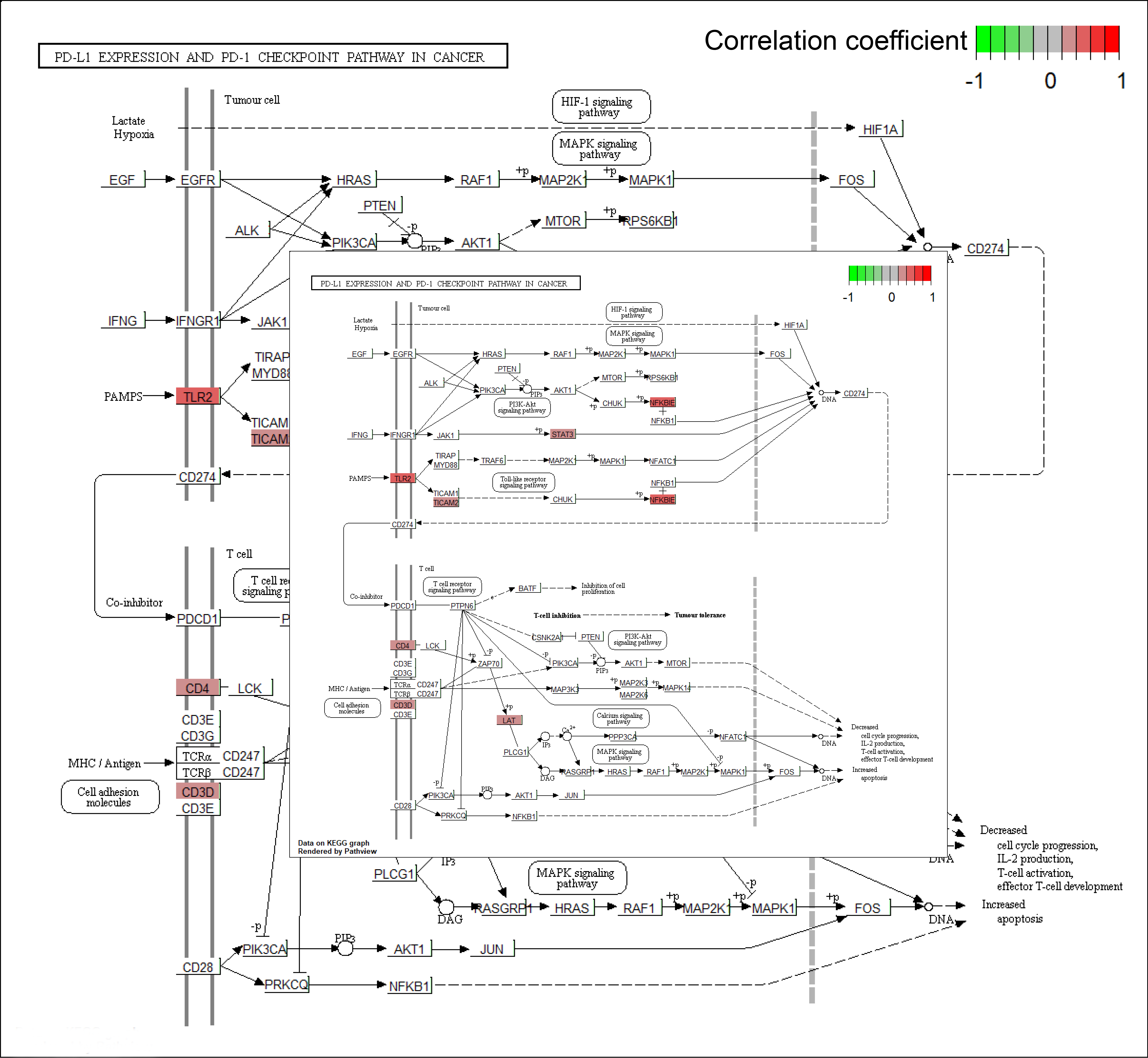

Supplement: Supplementary file 6 — Figure S6 [file CAM4-12-4938-s001.tif]

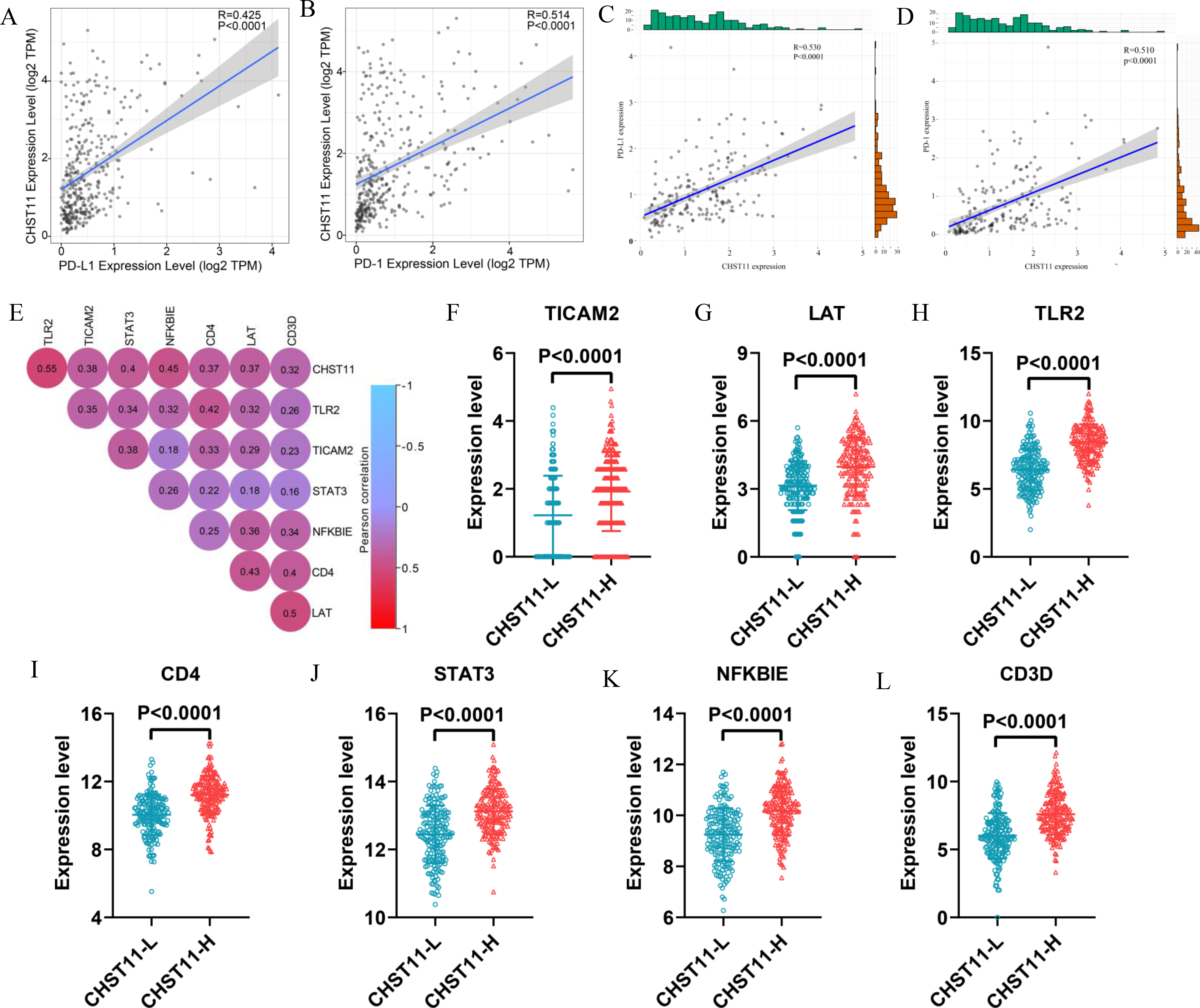

Supplement: Supplementary file 7 — Figure S7 [file CAM4-12-4938-s007.tif]
