# Supplementary material for: Highly expressed carbohydrate sulfotransferase 11 correlates with unfavorable prognosis and immune evasion of hepatocellular carcinoma
Source: Cancer Med. 2022 Sep 5;12(4):4938–50. doi: 10.1002/cam4.5186 (PMC9972111; doi:10.1002/cam4.5186)
Supplement: Supplementary file 8 — Table S1 [file CAM4-12-4938-s005.docx]

**Supplementary Table S1.** Relationship between CHST11 protein expression and clinicopathological parameters

| Clinicopathological parameters | Group | Number of cases | Z | P |
| --- | --- | --- | --- | --- |
| Age | ≤50 | 39 | 0.027 | 0.979 |
|  | >50 | 51 |  |  |
| Gender | Male | 46 | 1.478 | 0.14 |
|  | Female | 44 |  |  |
| Tumor size | ≤5cm | 49 | 0.041 | 0.967 |
|  | >5cm | 39 |  |  |
| Pathology grading | I-II | 49 | 1.36 | 0.174 |
|  | III-IV | 41 |  |  |
| BCLC stage | 0+A | 59 | 0.323 | 0.747 |
|  | B+C | 31 |  |  |

BCLC: Barcelona Clinic Liver Cancer
